# Deep learning model-assisted detection of kidney stones on computed tomography

**DOI:** 10.1590/S1677-5538.IBJU.2022.0132

**Published:** 2022-05-18

**Authors:** Alper Caglayan, Mustafa Ozan Horsanali, Kenan Kocadurdu, Eren Ismailoglu, Serkan Guneyli

**Affiliations:** 1 Izmir Bakırcay University Cigli Training and Research Hospital Department of Urology Izmir Turkey Department of Urology, Izmir Bakırcay University Cigli Training and Research Hospital, Izmir, Turkey; 2 Izmir Bakırcay University Cigli Training and Research Hospital Department of Information Systems Izmir Turkey Department of Information Systems, Izmir Bakırcay University Cigli Training and Research Hospital, Izmir, Turkey; 3 Izmir Bakırçay University Faculty of Medicine Deparment of Radiology Izmir Turkey Deparment of Radiology, Izmir Bakırçay University, Faculty of Medicine, Izmir, Turkey

**Keywords:** Kidney Calculi, Tomography, X-Ray Computed, Algorithms, Artificial Intelligence

## Abstract

**Introduction:**

The aim of this study was to investigate the success of a deep learning model in detecting kidney stones in different planes according to stone size on unenhanced computed tomography (CT) images.

**Materials and Methods:**

This retrospective study included 455 patients who underwent CT scanning for kidney stones between January 2016 and January 2020; of them, 405 were diagnosed with kidney stones and 50 were not. Patients with renal stones of 0–1 cm, 1–2 cm, and >2 cm in size were classified into groups 1, 2, and 3, respectively. Two radiologists reviewed 2,959 CT images of 455 patients in three planes. Subsequently, these CT images were evaluated using a deep learning model. The accuracy rate, sensitivity, specificity, and positive and negative predictive values of the deep learning model were determined.

**Results:**

The training group accuracy rates of the deep learning model were 98.2%, 99.1%, and 97.3% in the axial plane; 99.1%, 98.2%, and 97.3% in the coronal plane; and 98.2%, 98.2%, and 98.2% in the sagittal plane, respectively. The testing group accuracy rates of the deep learning model were 78%, 68% and 70% in the axial plane; 63%, 72%, and 64% in the coronal plane; and 85%, 89%, and 93% in the sagittal plane, respectively.

**Conclusions:**

The use of deep learning algorithms for the detection of kidney stones is reliable and effective. Additionally, these algorithms can reduce the reporting time and cost of CT-dependent urolithiasis detection, leading to early diagnosis and management.

## INTRODUCTION

Urolithiasis is a common health problem, with a worldwide prevalence of 1.7-14.8% (1). Multiple factors contribute to the increase in its prevalence, including lifestyle changes, nutritional habits, obesity, diabetes mellitus, metabolic syndrome, and hypertension. In the United States, more than 2 million people with renal colic are admitted to the emergency departments each year, and approximately half of these patients undergo unenhanced computed tomography (CT). From 1992 to 2009, the use of CT was estimated to triple in the United States, with a corresponding increase in its cost of use (2, 3). The cost of nephrolithiasis management is high for both individuals and the society. The choice of the most appropriate treatment for kidney stones is challenging because it depends on several factors such as the type, shape, size, and location of the stones (4).

Deep learning is a type of machine learning termed artificial neural networks and is inspired by the structure and function of the brain (5). Nowadays, with the successful use of computer vision with deep learning algorithms, deploying these algorithms to study medical images has become popular (6). Artificial intelligence (AI)-based systems for the evaluation of unenhanced CT images may be used to develop reliable and accurate anatomical models for operational support, as well as for predicting the success rate and outcomes of the treatment (7, 8). These systems assist medical decision-making and minimize iatrogenic errors in clinical practice. AI models employ synergistic working methods where learning abilities and performance are developed rather than a priori coded. Therefore, these models can fulfil their tasks with high speed, functionality, and efficiency (9). We hypothesized that AI can be efficiently used to diagnose and detect kidney stones. In the present study, we aimed to investigate the success of a deep learning model for the diagnosis of kidney stones.

## MATERIALS AND METHODS

### Study population

This study was approved by the ethics committee of our institution (permission number: 378/358; dated: 10/11/2021). For this retrospective study, we selected 455 patients, between January 2016 and January 2020, of whom 405 bore kidney stones diagnosed via CT while the remaining 50 did not. A total of 2,959 unenhanced CT images, including 2,709 with kidney stones and 250 without, were evaluated by two experienced abdominal radiologists (X.X. with 12 years of experience and Y.Y. with 8 years of experience) based on consensus and using a dedicated workstation. Kidney stone diagnoses were based on their observation in the renal collecting system and on the measurement of Hounsfield units on unenhanced CT images. The final diagnosis of kidney stones was made by a radiologist. The patients were divided into three groups as follows: group 1 contained patients with renal stone sizes of 0–1 cm, group 2 had sizes of 1-2 cm, and group 3 had sizes greater than 2 cm. When multiple kidney stones were present, the largest stone size was included in the study. The results of the AI algorithm for the detection of kidney stones were compared with the radiologists’ diagnoses to determine the efficiency of the AI model.

Patients with solitary kidneys, atrophic kidneys, renal anomalies, calcified renal masses, renovascular calcifications, regional lymph node calcifications, metallic implants, pigtail ureteral catheters, percutaneous nephrostomy catheters, and CT images with artefacts were excluded from the study.

### CT image acquisition

The anatomical area between the diaphragm and the symphysis pubis was scanned using a CT scanner (Optima CT 660, GE Healthcare System, Milwaukee, USA). During the scan, the gantry angle was set to 0°; the matrix size, 512×512 pixels; the voltage, 120 kV; the tube current; 100-200 mAs; the collimation, 64×0.5; and the slice thickness, ≤1.25 mm on a 128-slice CT device. All images were reconstructed in the axial, coronal, and sagittal planes with a 2-mm section thickness using the medical imaging program, AW Server 3.2 Ext. 1.2 by GE Healthcare.

### Artificial Intelligence Algorithm

**Figure 1:**
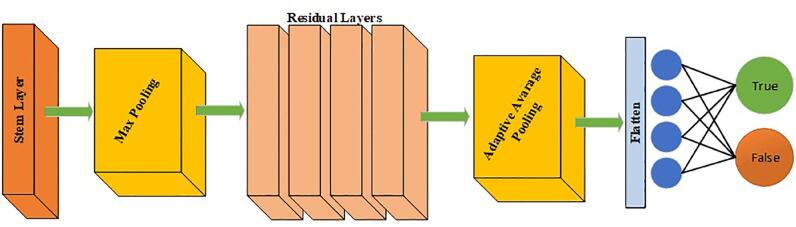
Layer organization of the xResNet50 deep learning model. xResNet50 architecture consists of an input stem, four xResNet50 blocks, and an output stem. Images are put in from the input stem, then processed in the model, and classified in the output stem; finally, they are returned as a percentage of kidney stone presence or absence.

ResNet is a convolution-based deep residual network architecture. ResNet consists of several residual blocks (composed of a convolutional layer), a batch normalization layer, and a shortcut that connects the original input to the output of the residual block (8). We used the xResNet50 convolutional neural network architecture in our study. The xResNet architecture was derived from the convolution-based deep residual network architecture ResNet with a few minor changes (7). The layer organization of the model is shown in Figure-1.

The number of patients and CT images with and without kidney stones in the three groups according to the sizes of kidney stones evaluated using the deep learning algorithm are presented in Table-1.

**Table 1:** Number of patients and CT images with and without kidney stones of 3 groups evaluated with the deep learning algorithm and the training group accuracy rates of the deep learning model of the CT images.

		Group 1 (0-1 cm)		Group 2 (1-2 cm)		Group 3 (>2 cm)	
		Patient n, %	Image n, %	Patient n, %	Image n, %	Patient n, %	Image n, %
**AI training**	**Normal**	40 (30.5%)	200 (21%)	40 (26.8%)	200 (21%)	40 (24.1%)	200 (21%)
	**Stone**	91 (69.5%)	753 (79%)	109 (73.2%)	753 (79%)	126 (75.9%)	753 (79%)
	**Accuracy**						
	Axial	98.2%		99.1%		97.3%	
	Coronal	99.1%		98.2%		97.3%	
	Sagittal	98.2%		98.2%		98.2%	
**AI testing**	Normal	10 (18.5%)	50 (25%)	10 (27.7%)	50 (25%)	10 (52.6%)	50 (25%)
	Stone	44 (81.5%)	150 (75%)	26 (72.3%)	150 (75%)	9 (47.4%)	150 (75%)
	**Accuracy**						
	Axial	78.0%		68.0%		70.0%	
	Coronal	63.0%		72.0%		64.0%	
	Sagittal	85.0%		89.0%		93.0%	

**AI** = Artificial Intelligence

First, training of the CT images of patients with and without kidney stones was performed using the AI model, followed by model testing. Training and testing were performed in the three planes and among the three study groups using the Fastai (v2) library and the Google Collaboratory platform. We used the Adam algorithm as the optimization algorithm (10). Cross-entropy loss was utilized as the loss function. During model training, we selected the learning rate to be 0.01 and achieved the best validation scores after an average of the 35th epoch. The images used for training the model were not preprocessed or augmented in any way (7–10).

### Statistical Analysis

All statistical analyses were conducted using SPSS Statistics version 26.0 (IBM Inc., Chicago, IL, USA). The demographic characteristics of the patients are presented as mean±standard deviation for continuous variables and as median and percentage for categorical variables. The sensitivity, specificity, and positive and negative predictive values of the results for all planes were calculated using receiver operating characteristic (ROC) curve analysis for each group.

## RESULTS

**Figure 2:**
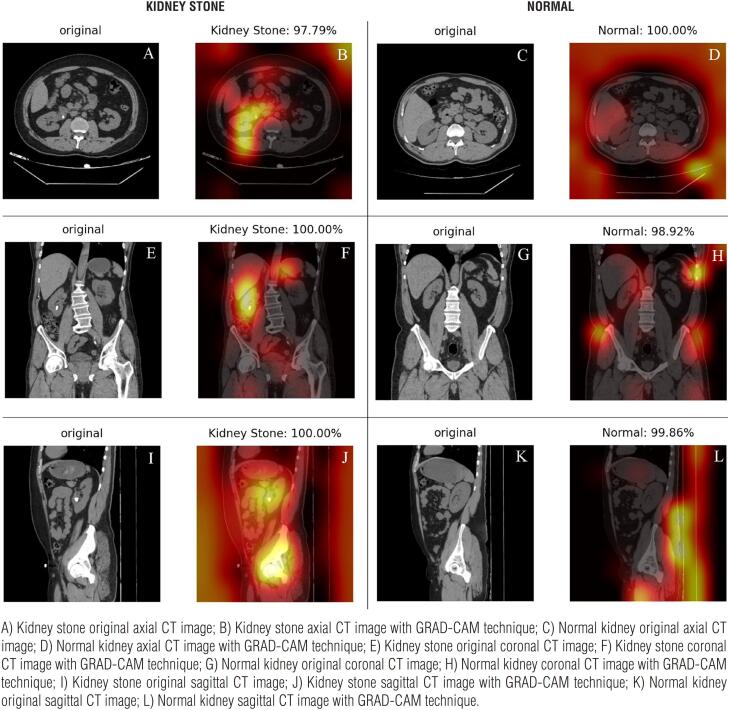
Axial, sagittal, and coronal CT images of patients bearing 0–1-cm-sized kidney stones and without kidney stones, demonstrated with the Grad-CAM technique. Percentages refer to the estimates of the AI model.

**Figure 3:**
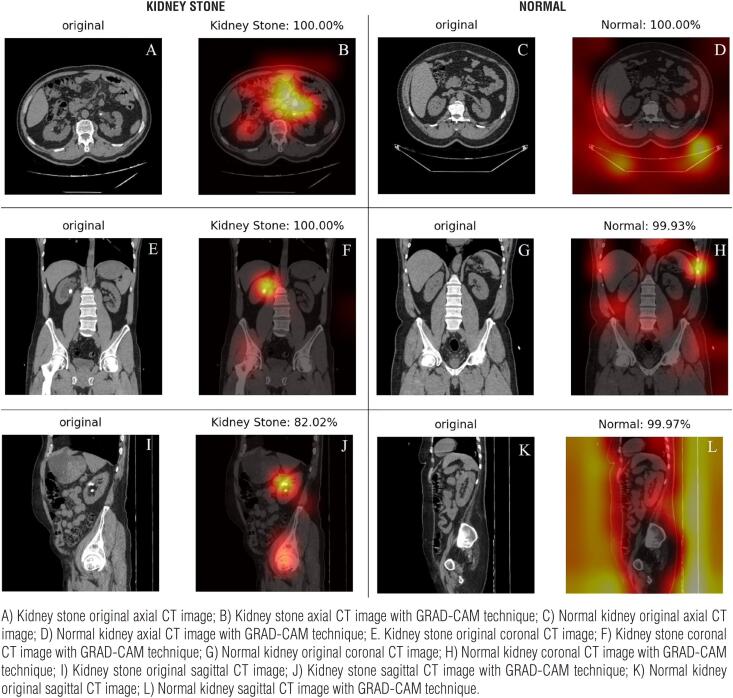
Axial, sagittal, and coronal CT images of the patients bearing 1–2 cm-sized kidney stones and without kidney stones, demonstrated with the Grad-CAM technique. Percentages refer to the estimates of the AI model.

**Figure 4:**
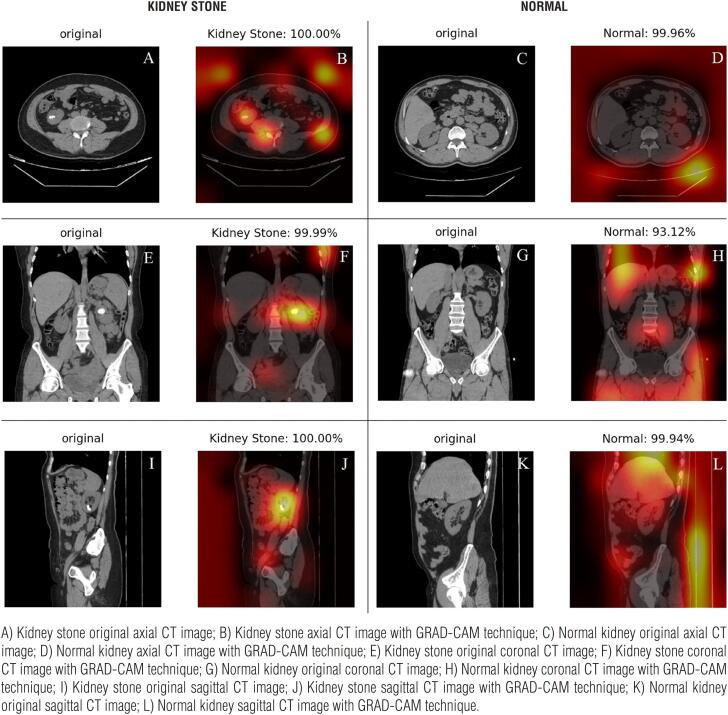
Axial, sagittal, and coronal CT images of the patients bearing kidney stones greater than 2 cm in size and without kidney stones, demonstrated with the Grad-CAM technique. Percentages refer to the estimates of the AI model.

The mean age of patients in our study was 42.54±14.76 (range: 23-77) years. Two hundred ninety-two (65.2%) patients were male and 163 (35.8%) were female. We used the Grad-CAM technique, which is deployed to produce “visual explanations” for convolutional neural networks, to identify the areas where our models were concentrated (11). CT images of the patients in the three study groups visualized with the Grad-CAM technique are demonstrated in Figures 2, 3, and 4.

The accuracy rates of the deep learning model in the training group are presented in Table-1. The success rates were as follows: 98.2% in the axial section, 99.1% in the coronal section, and 98.2% in the sagittal section in group 1; 99.1% in the axial section, 98.2% in the coronal section, and 98.2% in sagittal section in group 2; 97.3% in the axial section, 97.3% in the coronal section, and 98.2% in the sagittal section in group 3.

The AI accuracy rates for the three planes in the three groups are presented in Table-2. The success rates obtained by verifying the trained deep learning model in the test group were: 78% in the axial section, 63% in the coronal section, and 85% in the sagittal section in group 1; 68% in the axial section, 72% in coronal section and 89% in sagittal section in group 2; and 70% in the axial section, 64% in the coronal section, and 93% in the sagittal section in group 3.

**Table 2:** Accuracy rates, sensitivity, specificity, positive predictive, and negative predictive values of the deep learning model for the planes among 3 groups of kidney stones.

Classification Reports (Test Set)		Axial		Coronal		Sagittal	
		normal	stone	normal	stone	normal	stone
**Group 1 (0-1 cm)**	Precision	76.0 %	80.0 %	69.0 %	60.0 %	**83.0 %**	**87.0 %**
	Recall	82.0 %	74.0 %	48.0 %	78.0 %	**88.0 %**	**82.0 %**
	f1-score	79.0 %	77.0 %	56.0 %	68.0 %	**85.0 %**	**85.0 %**
	Accuracy	78.0 %		63.0 %		**85.0 %**	
	Positive Predictive Value	75.0 %		78.0 %		**82.0 %**	
	Negative Predictive Value	82.0 %		48.0 %		**88.0 %**	
	Sensivity	80.4 %		60.0 %		**87.2 %**	
	Specificity	75.9 %		68.5 %		**80.0 %**	
**Group 2 (1-2 cm)**	Precision	70.0 %	66.0 %	76.0 %	69.0 %	**91.0 %**	**87.0 %**
	Recall	62.0 %	74.0 %	64.0 %	80.0 %	**86.0 %**	**92.0 %**
	f1-score	66.0 %	70.0 %	70.0 %	74.0 %	**89.0 %**	**89.0 %**
	Accuracy	68.0 %		72.0 %		**89.0 %**	
	Positive Predictive Value	74.0 %		80.0 %		**92.0 %**	
	Negative Predictive Value	62.0 %		64.0 %		**86.0 %**	
	Sensivity	66.1 %		68.9 %		**86.7 %**	
	Specificity	70.4 %		76.1 %		**91.4 %**	
**Group 3 (>2 cm)**	Precision	73.0 %	68.0 %	85.0 %	59.0 %	**94.0 %**	**92.0 %**
	Recall	64.0 %	76.0 %	34.0 %	94.0 %	**92.0 %**	**94.0 %**
	f1-Score	68.0 %	72.0 %	49.0 %	72.0 %	**93.0 %**	**93.0 %**
	Accuracy	70.0 %		64.0 %		**93.0 %**	
	Positive Predictive Value	76.0 %		94.0 %		**94.0 %**	
	Negative Predictive Value	64.0 %		34.0 %		**92.0 %**	
	Sensivity	67.8 %		58.7 %		**92.1 %**	
	Specificity	72.7 %		85.0 %		**93.8 %**	

The sensitivity, specificity, and positive and negative predictive values of the AI algorithm for the planes in the three groups are presented in Table-2.

## DISCUSSION

In our study, we investigated the success rate of AI methodologies in the diagnosis of kidney stones and found that the AI-based system we used provided accurate results. The sensitivity and specificity of diagnosis based on sagittal plane images were found to be higher than those of the other planes. This study is the first study in the literature to use an artificial intelligence model in the diagnosis of urinary system stone disease by classifying both in 3 different imaging axes and according to different stone sizes.

In a study by Imamura et al., choosing an appropriate imaging modality for the diagnosis of stones resulted in a high stone-free rate, low morbidity, high probability of survival, fast recovery, and low treatment cost (12). The guidelines provided by the American College of Radiology, the American Urological Association, and the European Association of Urology differ in the optimal initial imaging modality being used for evaluating patients with suspected obstructive nephrolithiasis. Although CTs of the abdomen and pelvis provide the most accurate diagnosis, they expose patients to harmful ionizing radiations. Ultrasonography has lower sensitivity and specificity than CT but does not require the use of radiation. Radiography of the kidney, ureter, and bladder is very helpful in the periodic evaluation of stone growth in patients with known stone disease but has limited utility in the diagnosis of acute stones. Of all the imaging modalities available currently, CT is the most sensitive technique for detecting kidney stones with a sensitivity of approximately 95% (13).

Cost and reimbursement issues among CT stakeholders, including hospitals, insurance companies, and patients, often complicate the choice of CT as an imaging modality. A review of Medicare data revealed that the cost of performing a CT scan is approximately double that of a renal ultrasound scan and approximately one third that of an MRI. This has caused AI models to come to the forefront in terms of cost, efficiency, and imaging preference (14, 15).

Recently, artificial neural network-based AI has attracted significant attention in medical imaging. An artificial neural network (ANN) calculates the output value from multiple input values using a simple mathematical neuron model. ANN systems are composed of a large number of neurons arranged in interconnected layers that can be trained to predict results based on the input of the first layer. In contrast, conventional neural networks have convolutional layers that are suitable for image analysis. Conventional neural networks can be fed with annotated images and can learn classification with automatic iterative adjustments of weighted neural functions (16, 17).

Computer-aided detection/diagnosis (CADe/CADx) is a successful research area in medical image processing. Recent developments have revealed the importance of applying conventional neural network-based deep learning algorithm approaches, although they require a large amount of training data (16, 18). Yan et al. developed a universal lesion detector (DeepLesion) that can detect any lesion with a single unified frame (19).

The use of AI in urology has considerably increased in recent years. In particular, studies comparing AI models with imaging methods in diagnosis and patient selection have been reported. Recently, there has been an increase in the demand for CT in the diagnosis of kidney stones due to an increase in the number of patients suffering from this condition. This has led to a prolongation of the radiological evaluation period owing to the relatively less number of radiologists available to evaluate the images (20). Furthermore, during the coronavirus disease pandemic, reporting processes have become even more problematic due to the increased workload of radiologists. This workload also resulted in reducing surgery volumes and urology residency programs (21, 22). In such a scenario, using computer-assisted AI methods to diagnose urolithiasis can ensure a fast and accurate diagnosis, leading to early management in urological clinical practice.

Längkvist et al. developed a conventional neural-network method to detect ureteral stones in thin-section CT scans and showed that CT images can be read primarily with an automated detection algorithm (23). Sokolovskaya et al. found a significant positive relationship between the fast-reading speed of tomography and the number of interpretation errors. Furthermore, several studies reported that diagnostic errors due to radiological diagnosis maybe due to perceptual and cognitive interpretation errors of radiologists and that strategies to improve the performance of radiologists should be developed (12, 24, 25). Another study revealed that developing a machine learning-based system can assist urologists in managing large kidney stones (26). Recent technological advances have demonstrated high sensitivity, specificity, and positive predictive value in detecting urinary tract stones ≥3 mm with an average radiation dose of 1-1.5 mSv, allowing for dose reduction with the advent of low-dose CT techniques (27).

Our study bears several limitations. The major limitation of this study was the lack of consideration of the stone composition, which is one of the most important parameters in the management of kidney stones. Another limitation was the lack of testing of the effect of the AI algorithm in predicting the success of the treatment.

## CONCLUSIONS

Deep learning models are reliable and effective for the detection of kidney stones. The sagittal-plane images on CT had higher diagnostic accuracy rates than those of other planes. Using these methods, the waiting time for results and cost of diagnosis can be reduced, and early diagnosis can be achieved, resulting in prompt management.
